# Reduced perinatal mortality following enhanced training of birth attendants in the Democratic Republic of Congo: a time-dependent effect

**DOI:** 10.1186/1741-7015-9-93

**Published:** 2011-08-04

**Authors:** Richard Matendo, Cyril Engmann, John Ditekemena, Justin Gado, Antoinette Tshefu, Rinko Kinoshita, Elizabeth M McClure, Janet Moore, Dennis Wallace, Waldemar A Carlo, Linda L Wright, Carl Bose

**Affiliations:** 1 Kinshasa School of Public Health, Kinshasa, Democratic Republic of Congo; 2 Division of Neonatal-Perinatal Medicine, CB#7596, University of North Carolina at Chapel Hill, North Carolina 27599-7596, USA; 3 Research Triangle Institute, PO Box 12194, Research Triangle Park, NC 27709, USA; 4 Women & Infants Center, University of Alabama at Birmingham, 1700 6th Ave South, Birmingham, AL 35233, USA; 5 Eunice Kennedy Shriver National Institute of Child Health and Human Development, Building 31, Room 2A32, MSC 2425, 31 Center Drive, Bethesda, Maryland 20892-2425, USA

## Abstract

**Background:**

In many developing countries, the majority of births are attended by traditional birth attendants, who lack formal training in neonatal resuscitation and other essential care required by the newly born infant. In these countries, the major causes of neonatal mortality are birth asphyxia, infection, and low-birth-weight/prematurity. Death from these causes is potentially modifiable using low-cost interventions, including neonatal resuscitation training. The purpose of this study was to evaluate the effect on perinatal mortality of training birth attendants in a rural area of the Democratic Republic of Congo (DRC) using two established programs.

**Methods:**

This study, a secondary analysis of DRC-specific data collected during a multi-country study, was conducted in two phases. The effect of training using the WHO Essential Newborn Care (ENC) program was evaluated using an active baseline design, followed by a cluster randomized trial of training using an adaptation of a neonatal resuscitation program (NRP). The perinatal mortality rates before ENC, after ENC training, and after randomization to additional NRP training or continued care were compared. In addition, the influence of time following resuscitation training was investigated by examining change in perinatal mortality during sequential three-month increments following ENC training.

**Results:**

More than two-thirds of deliveries were attended by traditional birth attendants and occurred in homes; these proportions decreased after ENC training. There was no apparent decline in perinatal mortality when the outcome of all deliveries prior to ENC training was compared to those after ENC but before NRP training. However, there was a gradual but significant decline in perinatal mortality during the year following ENC training (RR 0.73; 95% CI: 0.56-0.96), which was independently associated with time following training. The decline was attributable to a decline in early neonatal mortality. NRP training had no demonstrable effect on early neonatal mortality.

**Conclusion:**

Training DRC birth attendants using the ENC program reduces perinatal mortality. However, a period of utilization and re-enforcement of training may be necessary before a decline in mortality occurs. ENC training has the potential to be a low cost, high impact intervention in developing countries.

**Trial registration:**

This trial has been registered at http://www.clinicaltrials.gov (identifier NCT00136708).

## Background

More than 98% of the estimated 3.7 million neonatal deaths and 3.2 million stillbirths per year occur in developing countries[1]. In many of these countries, the majority of deliveries occur in homes, either unattended by any health care provider or attended by Traditional Birth Attendants (TBAs)[2]. For example, in rural areas of the Democratic Republic of Congo (DRC), more than three- fourths of births are attended by TBAs or a family member[3]. TBAs often lack formal training in neonatal resuscitation, and other essential care required by the newly born infant.

The major causes of neonatal mortality are birth asphyxia, infection, and low-birth-weight/prematurity [4,5], and death from these causes is potentially modifiable. Low-cost interventions, including neonatal resuscitation training [6], may decrease neonatal deaths by up to 50%[7-9]. In a recent study, the FIRST BREATH Trial, training in the World Health Organization (WHO) Essential Newborn Care (ENC) program reduced stillbirths by 30% without increasing early neonatal mortality among women delivering in six developing countries[10]. Our study group in the DRC participated in the FIRST BREATH Trial.

Features of the perinatal care systems that might have influenced birth outcomes, for example, type of birth attendant, location of births and support systems, varied among sites in the FIRST BREATH Trial. Our objective was to evaluate outcomes following training of birth attendants in the unique context of the environment in the DRC by evaluating the effect of training on rates of stillbirth, early neonatal (seven days or less of age) and perinatal mortality in fetuses/infants weighing ≥1,500 grams at birth.

## Methods

This study is a secondary analysis of data collected during the FIRST BREATH Trial, a multi-country study conducted by the Eunice Kennedy Shriver National Institute of Child Health and Human Development Global Network for Women's and Children's Health Research[10]. That study was a population-based, prospective interventional study conducted in two phases. In the first phase, the impact of training using the ENC program was evaluated using an active baseline design. A period of prospective data collection was followed by ENC training and continuation of data collection. This phase was followed by a cluster randomized trial of training using an adaptation of the Neonatal Resuscitation Program (NRP: American Academy of Pediatrics and American Heart Association; 2000 edition). Communities were randomized to either receive NRP training (intervention communities) or to continue to provide care without additional training (control communities).

The study was conducted in the rural Equateur Province, where the economy is based on subsistence farming. Women are active participants in family farms; few work in other occupations. More than one-third of the population is in the lowest economic quintile of the country. Communities were selected to be representative of this province. Each had at least 300 births per year and was geographically distinct, such that the birth attendants in one community did not attend deliveries in another community. Most communities had poor health systems with a high rate of home births assisted by TBAs. The study in the DRC was approved by the institutional review boards at the Kinshasa School of Public Health and the University of North Carolina. Informed consent was obtained from the mothers.

The study included all births in 12 communities, including deliveries in hospitals, health clinics and homes. Pregnant women were enrolled at their first antenatal visit or at the onset of labor for those who had not received antenatal care. Data describing maternal, pregnancy, delivery, and neonatal variables and exposures, and seven-day outcomes were collected. Data analyses were limited to fetuses/neonates with birth weights ≥1,500 grams (or appearing to be ≥1,500 grams if weight was not obtained).

### Training procedures

A train-the-trainer educational program was developed using a variety of teaching methods, including clinical practice sessions and demonstrations. Supervisory personnel of the FIRST BREATH Trial trained two DRC Country Trainers. The supervisory personnel had extensive global health and educational experience, and specifically in instruction in both the ENC program and NRP. Country Trainers were general medical doctors with experience in rural health practice but no previous training in the ENC program or NRP. In a five-day workshop, the Country Trainers subsequently trained one Community Coordinator (CC), a professionally trained nurse, from each study community and the four physicians who staffed the district hospitals. In addition to instruction in the program content, these trainees were taught adult education methodologies. Thereafter, in a series of three-day workshops (one for each phase of the study; see below), the CCs and local physicians trained the practicing birth attendants within each community. Birth attendants included 152 TBAs and 18 nurses and nurse midwives. Nurses in the DRC typically complete four years of nursing education following ten years of primary and secondary education. Nurse midwives receive an additional three years of maternal and child health education. TBAs receive variable training, which is usually limited to an apprenticeship with an experienced TBA. They do not have formal education and are usually illiterate. Separate workshops were conducted for TBAs and for nurses and midwives. All workshops for each phase of the study were completed within one calendar month.

The study began with training in data collection and other basic study procedures, including evaluation of newborns, particularly the identification of vital signs to ensure the proper differentiation between stillbirths and live born infants. Scales for weighing newborns (Salter Scales; UNICEF model 145555) and disposable delivery kits (Population Services International, Washington, DC) were distributed at this time. Thereafter, a period of baseline data collection began.

After the period of baseline data collection, the train-the-trainer model was used to train all personnel in the ENC program[11]. The ENC program content included routine neonatal care, initiation of breathing and resuscitation, thermoregulation, early and exclusive breastfeeding, kangaroo (skin-to-skin) care, small baby care, recognition of danger signs, and recognition and initial management of complications. Resuscitation training was limited to recognizing the apneic infant and the use of stimulation and manual ventilation. Ventilation bags and face masks were distributed, and instruction in their use was provided. Educational materials, utilizing drawings in lieu of text, were developed for training of TBAs.

After an additional period of data collection, birth attendants in six randomly selected communities received more extensive resuscitation training using an adaptation of the NRP. This training also utilized a train-the-trainer model, and material that was adapted for the environment. This intensive basic resuscitation course included the initial steps of resuscitation and bag and mask ventilation, but excluded the use of oxygen, intubation, chest compression and the administration of drugs. In contrast to the resuscitation training in the ENC program, NRP training included a systematic approach to resuscitation that included repeated observations throughout the first few minutes following delivery and standardized responses to these observations. A refresher course was conducted six months after initial training.

Reinforcement of training occurred throughout the study at monthly meetings attended by CCs and birth attendants in each community. The CCs (the original trainers of the birth attendants) reviewed the ENC program in all communities. In addition, they reviewed the NRP in the intervention communities. The content of the meetings was left to the discretion of the CC, but in general was based on observations made during the supervision of the birth attendants' activities, including deliveries when possible. Deficiencies in care or gaps in knowledge were the focus of discussions at these meetings. Additional training was usually practical rather than theoretical and involved repetition of hands-on skills, for example, mannequins and bags and masks were used to practice resuscitation skills. Although we did not systematically record attendance, we estimate that more than 95% of birth attendants participated in these meetings.

### Data collection and management

CCs or birth attendants obtained consent (at the time of enrollment) and collected all data on standardized data forms. Community Health Workers assisted illiterate TBAs in the completion of these forms. Data forms were reviewed for accuracy by the CCs. Data edits, including inter- and intra-form consistency checks, were performed upon data entry locally and by the data coordinating center (RTI International, Research Triangle Park, NC, USA).

### Sample size, study outcomes and statistical analyses

The number of communities selected from the DRC was determined by the sample size calculation for the FIRST BREATH Trial, and the number of deliveries observed was a function of the timing of the study periods for that trial[10].

The primary outcome for the analyses in this manuscript was perinatal mortality (stillbirth or death during the first seven days after birth). We used this combined outcome, in lieu of using early neonatal mortality, to eliminate the possibility that early neonatal deaths incorrectly classified as stillbirths would confound the results. Pre-specified secondary outcomes included stillbirth and early neonatal mortality rates, mortality rates stratified by sex, birth weight, birth location and birth attendant, and one and five minute Apgar scores and the use of bag/mask ventilation.

Generalized estimating equation (GEE) extensions of logistic regression models that adjust for correlation of outcomes within communities were used to determine differences in maternal and neonatal characteristics between the pre- and post-ENC data and between the NRP intervention and control data. Adjusted relative risks (RR) and 95% confidence intervals (CI) using GEE extensions of multivariate log-binomial models are reported for the post- versus the pre- comparison of the effect of training in ENC, in the comparison of the NRP intervention versus control (no NRP) groups, and in the comparison of post-NRP versus pre-ENC. The data were analyzed using SAS version 9.2 (Cary, NC). To examine the possibility that benefits of ENC training were time dependent, we analyzed perinatal mortality as a function of time, modeled continuously, using a robust piecewise Poisson GEE model that included two covariates, location of birth and birth attendant.

## Results

### Comparison of outcomes from baseline to post- ENC training

**Figure 1:**
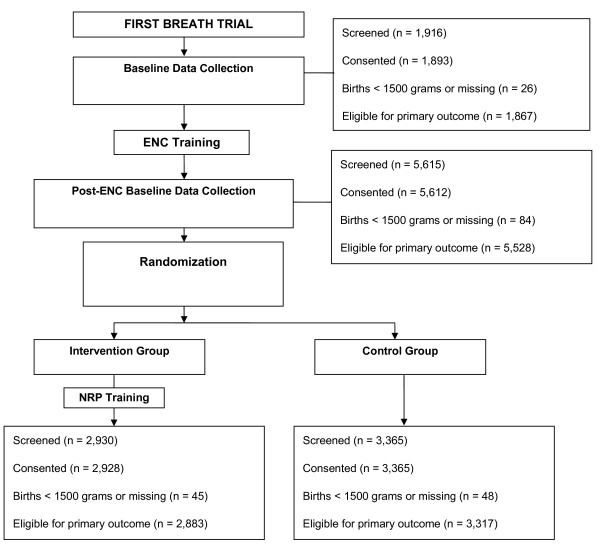
**Derivation of the study population**.

The study was conducted in 12 communities. Baseline data were collected from June of 2005 until November 2005 in all but two communities; in these communities, the study did not begin until July of 2006, and there was no period of baseline data collection. Therefore, only post-NRP data from these communities (one control and one intervention community) were used in analyses. During pre-ENC period, there were 1,867 births, and following ENC training but before NRP training, a period of approximately 13 months, there were 5,528 births (Figure 1 and Table 1). During the baseline pre-ENC period, nearly 80% of births were attended by TBAs, and a similar proportion occurred either in the home of the mother or of the TBA. Following ENC training, more births were attended by nurses or nurse midwives, and births more commonly occurred in clinics. The proportion of Apgar scores <4 at one and five minutes and the use of bag and mask ventilation remained the same after ENC training compared to during the baseline period.

**Table 1:** Neonatal and demographic characteristics

	Pre-ENC (N = 1,867)	Post-ENC (N = 5,528)	*P*-value	Post-NRP Control (N = 3,317)	Post-NRP Intervention (N = 2,883)	*P*-value
**Birth attendant**^**a**^	1,867	5,528	0.0011	3,317	2,883	0.9971
Physician	5 (0.3)	1 (0.0)		1 (0.0)	1 (0.0)	
Nurse/midwife	330 (17.7)	1,242 (22.5)		973 (29.3)	859 (29.8)	
Traditional birth attendants	1,481 (79.3)	4,248 (76.8)		2,331 (70.3)	2,012 (69.8)	
Family/unattended/other	51 (2.7)	37 (0.7)		12 (0.4)	11 (0.4)	
**Location of birth**^b^	1,867	5,528	0.0013	3,317	2,883	0.8681
Hospital	2 (0.1)	3 (0.1)		3 (0.1)	1 (0.0)	
Clinic	403 (21.6)	1,473 (26.6)		1,056 (31.8)	988 (34.3)	
Birth attendant home	1,328 (71.1)	4,019 (72.7)		2,248 (67.8)	1,889 (65.5)	
Home	116 (6.2)	13 (0.2)		4 (0.1)	4 (0.1)	
Other	18 (1.0)	20 (0.4)		6 (0.2)	1 (0.0)	
**Maternal education**	1,864	5,515	0.9603	3,315	2,880	0.0411
No school	1,204 (64.6)	3,545 (64.3)		2,472 (74.6)	1,693 (58.8)	
Primary	567 (30.4)	1,726 (31.3)		736 (22.2)	1,064 (36.9)	
Secondary or above	93 (5.0)	244 (4.4)		107 (3.2)	123 (4.3)	
**Neonatal characteristics**						
Multiple birth	55 (2.9)	176 (3.2)	0.7293	99 (3.0)	78 (2.7)	0.6131
Gender	1,857	5,522	0.1936	3,316	2,877	0.0382
Male	948 (51.1)	2,896 (52.4)		1,750 (52.8)	1,459 (50.7)	
Female	909 (48.9)	2,626 (47.6)		1,566 (47.2)	1,418 (49.3)	
Birth weight	1,853	5,508	0.7716	3,313	2,875	0.9781
1500-1999 grams	50 (2.7)	106 (1.9)		58 (1.8)	48 (1.7)	
2000-2499 grams	132 (7.1)	454 (8.2)		273 (8.2)	238 (8.3)	
≥ 2500 grams	1,671 (90.2)	4,948 (89.8)		2,982 (90.0)	2,589 (90.1)	
Apgar 1 min < 4	74/1,852 (4.0)	196/5,522 (3.5)	0.2625	105/3,316 (3.2)	85/2,877 (3.0)	0.7124
Apgar 5 min < 4	49/1,853 (2.6)	144/5,522 (2.6)	0.9186	91/3,316 (2.7)	67/2,877 (2.3)	0.5754
Apneic at birth	186/1,855 (10.0)	394/5,518 (7.1)	0.0017	193/3,315 (5.8)	158/2,877 (5.5)	0.7470
Bag and mask ventilation	1/1,867 (0.1)	14/5,528 (0.3)	0.1901	62/3,317 (1.9)	103/2,883 (3.6)	0.0793

Notes: Numbers in cells indicate number of infants for which data are available; percentages of the total are in parentheses. *P*-values are adjusted for cluster using GEE modeling. ^a^The **P**-values for birth attendants exclude physicians since the number of physician-attended deliveries is very small; ^b^The *P*-values for location of birth exclude hospital deliveries for the ENC period and hospital, home and other locations for the post-NRP period since the number of deliveries in these locations are very small.

Abbreviations: ENR, Essential Newborn Care; GEE, generalized estimating equation; NPR, Neonatal Resuscitation Program.

Outcome data at 7-days were available for 99.8% of births. The stillbirth, early neonatal and perinatal mortality rates did not decrease among all births during the period from ENC training to NRP training compared to births during the baseline pre-ENC period (Table 2), nor did these rates decrease during this time in any pre-specified subgroup defined by birth attendant, site of delivery or birth weight category (data not shown). To eliminate the possibility of bias due to differential exclusion of cases based on an estimated (rather than measured) birth weight less than 1,500 g, we performed another analysis limited to births in which birth weight was measured; results were materially unchanged (data not shown)

**Table 2:** Mortality Rates Before and After Training in the Essential Newborn Care Neonatal Resuscitation Programs

	Pre-ENC n (Rate/1000)	Post-ENC n (Rate/1000)	RR (95% CI) Post- vs. Pre-ENC	Post-NRP Intervention n (Rate/1000)	Post-NRP Control n (Rate/1000)	RR (95% CI) Intervention vs. Control
7-day neonatal mortality^a^	50 (27)	153 (28)	1.03 (0.76,1.41)	44(16)	56(17)	0.90 (0.47,1.72)
Stillbirth^b^	44 (24)	121 (22)	0.93 (0.68,1.26)	65 (23)	85 (26)	0.88 (0.48,1.61)
Perinatal mortality	94 (50)	274 (50)	0.99 (0.77,1.27)	109 (38)	141 (43)	0.89 (0.63,1.25)

^a^Rate per 1000 live births; ^b^Rate per 1000 total births. Abbreviations: ENC = Essential Newborn Care; NRP = Neonatal Resuscitation Program

### Comparison of outcomes after NRP training in intervention and control communities

Training in NRP occurred in January of 2007 in six communities, followed by a 12 to 15 month period of data collection in all 12 communities. There were no significant differences in rates of stillbirth, perinatal mortality and early neonatal mortality after the time of training in the NRP communities compared to the control communities (Table 2). There were no significant differences in mortality between communities in any of the subgroups defined by birth attendant, site of delivery or other variables.

### Comparison of outcomes as a function of time

We examined change in outcomes over the entire study period by comparing early neonatal and perinatal mortality rates in the pre-ENC cohort to rates in the cohort after NRP training. Because the mortality outcomes were not significantly different between control and intervention communities following NRP training, their data were combined in these analyses. There was a decline in perinatal mortality (50 to 40 per 1000 births; RR 0.8, 95% CI 0.66-0.97; Table 3) that was attributable to a decline in early neonatal mortality (27 to 17 per 1000 live births; RR 0.60, 95% CI 0.39-0.93; Table 4). The numbers-needed-to-treat for all birth attendants, that is the estimated numbers of infants that would need to be delivered following ENC training, to prevent one perinatal and one early neonatal death were 98 and 88, respectively. The declines in mortality occurred among births attended by all birth attendant types and both in homes and facilities, but the greatest reductions appeared to occur among deliveries attended by nurses or nurse midwives, and in clinics.

**Table 3:** Perinatal Mortality during the Pre-ENC Compared to the Post-NRP Periods of the Study

	Pre-ENC^a^	Post-NRP^a^	RR (95% CI)^b^
Perinatal mortality	94/1,867 (50)	250/6,185 (40)	0.80 (0.66, 0.97)
Male	52/948 (55)	152/3,202 (47)	0.87 (0.69, 1.09)
Female	42/909 (46)	95/2,976 (32)	0.69 (0.48, 0.99)
Birth attendant			
All birth attendants	91/1,816 (50)	246/6,162 (40)	0.80 (0.66, 0.97)
Physician	1/5 (200)	0/2 (0)	--
Nurse/midwife	25/330 (76)	85/1,827 (47)	0.61 (0.36, 1.04)
Traditional birth attendants	65/1,481 (44)	161/4,333 (37)	0.85 (0.66, 1.09)
Family/unattended/other	3/51 (59)	4/23 (174)	2.96 (0.77, 11.29)
Location of birth			
Home/birth attendant home	64/1,462 (44)	155/4,142 (37)	0.85 (0.66, 1.11)
Clinic	29/403 (72)	94/2,039 (46)	0.64 (0.42, 0.98)
Hospital	1/2 (500)	1/4 (250)	0.50 (0.08, 3.13)
Birth weight			
1500-1999 grams	22/50 (440)	40/106 (377)	0.86 (0.57, 1.29)
2000-2499 grams	19/132 (144)	59/509 (116)	0.81 (0.53, 1.22)
≥2500 grams	50/1,671 (30)	143/5,558 (26)	0.86 (0.66, 1.12)

^a^Numbers in cells represent the number of perinatal deaths in that category divided by the total number for which data was available. Perinatal mortality rate (deaths/1000 births) is in parentheses. ^b^Relative Risks (RR) and 95% confidence intervals (95% CI) are adjusted for cluster using GEE modeling. Abbreviations: ENR, Essential Newborn Care; GEE, generalized estimating equation; NPR, Neonatal Resuscitation Program.

**Table 4:** Seven-Day Neonatal Mortality during the Pre-ENC Compared to the Post-NRP Periods of the Study

	Pre-ENC^a^	Post-NRP^a^	RR (95% CI) ^b^
Seven-day neonatal mortality	50/1,823 (27)	100/6,035 (17)	0.60 (0.39, 0.93)
Male	25/921 (27)	63/3,113 (20)	0.75 (0.45, 1.23)
Female	25/892 (28)	36/2,917 (12)	0.44 (0.27, 0.72)
Birth attendant			
All birth attendants	49/1,774 (28)	98/6,014 (16)	0.59 (0.38, 0.91)
Physician	0/4 (0)	0/2 (0)	--
Nurse/midwife	14/319 (44)	32/1,774 (18)	0.41 (0.18, 0.92)
Traditional birth attendants	35/1,451 (24)	66/4,238 (16)	0.65 (0.37, 1.13)
Family/unattended/other	1/49 (20)	2/21 (95)	4.67 (0.90, 24.20)
Location of birth			
Home/birth attendant home	35/1,433 (24)	59/4,046 (15)	0.60 (0.36, 0.99)
Clinic	15/389 (39)	41/1,986 (21)	0.54 (0.26, 1.10)
Hospital	0/1 (0)	0/3 (0)	--
Birth weight			
1500-1999 grams	14/42 (333)	21/87 (241)	0.72 (0.34, 1.52)
2000-2499 grams	12/125 (96)	21/471 (45)	0.46 (0.25, 0.88)
≥2500 grams	24/1,645 (15)	56/5,471 (10)	0.70 (0.33, 1.50)

^a^Numbers in cells represent the number of neonatal deaths in that category divided by the total number for which data was available. Neonatal mortality rate (deaths/1000 live births) is in parentheses. ^b^Relative Risks (RR) and 95% confidence intervals (95% CI) are adjusted for cluster using GEE modeling. Abbreviations: ENR, Essential Newborn Care; GEE, generalized estimating equation; NPR, Neonatal Resuscitation Program.

**Figure 2:**
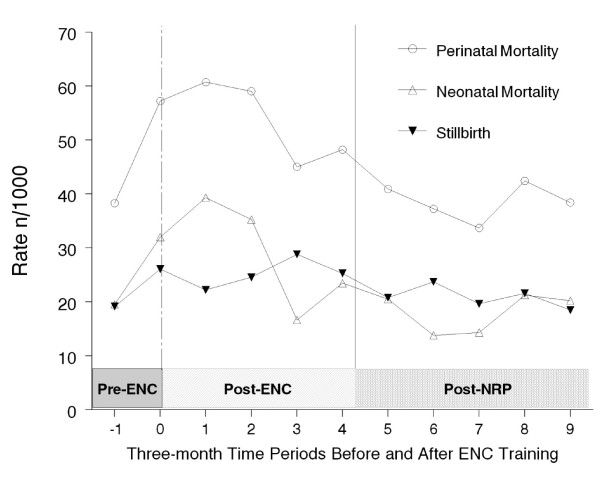
**Change in rates of stillbirth, and neonatal and perinatal mortality as a function of time during the study period**. Each time point represents the rates for the preceding three month period of time. The vertical lines indicate the time of ENC () and NRP training (). There was a significant decline in perinatal mortality during the year following ENC training, after adjustment for type of birth attendant and location of birth.

We explored the possibility that time following ENC training or other variables (for example, location of birth and birth attendant) might explain the reduction in perinatal mortality over the entire study period. We began by plotting mortality rates as a function of three-month increments of time (Figure 2). During the three months prior to ENC training, perinatal mortality appeared to increase because of increases in both stillbirths and neonatal mortality. At approximately three months after ENC training both perinatal and neonatal mortality appeared to decline. The piecewise Poisson model with a hinge at one year after ENC training showed that the coefficient for time since ENC training, after adjustment for birth attendant type and location of birth, was significant (*P *= 0.0235), and the relative risk of perinatal mortality for babies born one year after ENC training compared to those born at the start of ENC training was 0.73 (CI: 0.56-0.96). For the piece following the hinge point (one year after training) until the end of the study, there was not a significant correlation between time and mortality.

## Discussion

The FIRST BREATH Trial, a large multi-national study, investigated the effects of training birth attendants using the WHO ENC program and an adaptation of the AAP NRP[10]. Across all sites, ENC training resulted in a reduction in perinatal mortality when delivery was conducted by a birth attendant. There was no additional reduction following NRP training. Among countries in the study, there was marked variability in medical resources. For example, in the DRC, infants were rarely delivered by physicians, and less than 25% were delivered by medical professionals, compared to Argentina where most deliveries occurred in hospitals attended by physicians or nurse midwives (personal communication). We hypothesized that features of the environment within each country might have influenced the impact of these training programs on perinatal outcome. Therefore, our objective was to evaluate outcomes following training in the unique context of the environment in the DRC.

There was no apparent decline in rates of early neonatal and perinatal mortality or stillbirth when rates among all births during a baseline period of data collection prior to ENC training were compared to rates among births after ENC training but before NRP training, suggesting that ENC training was ineffective at reducing mortality. However, three observations caused us to examine the relationship between ENC training and mortality in an alternate way. First, perinatal mortality declined significantly over the entire study period. Second, mortality rates increased during the period of baseline data collection. The cause of this increase is not certain. However, a possible explanation is that some deaths were unreported at the outset of the study, and reporting became more reliable with time as a result of study oversight. Therefore, combining all births during the baseline period did not reflect the true baseline mortality rates; a more accurate estimate was more likely to be the rate during the latter portion of the baseline period. Finally, there was a gradual but significant decline in perinatal mortality during the first year following ENC training. Therefore, we examined change in mortality following ENC training as a function of time, after adjusting for known factors that might have confounded mortality rates (that is type of birth attendant and location of birth). After adjustment for these factors, a significant decline in perinatal mortality was associated independently with time during the first year following ENC training.

We are not aware of changes in any factors, such as birth rate or maternal mortality rate, other than type of birth attendant or location of birth that might have influenced perinatal outcomes during the conduct of the study. Therefore, we presume that the decline over time resulted from ENC training. One possible explanation for the time-dependency of benefit of ENC training is that repeated experience using the techniques included in the ENC program and/or re-enforcement of the education is necessary before implementation and improvement occurs. However, it is also possible that the decline in mortality resulted from changes in medical care not attributable directly to ENC training that were not recorded during the study, and therefore not accounted for in our analyses. These changes might include the availability of healthcare services, such as medications, diagnostic capabilities or transportation to healthcare facilities,

As was observed in the across-country analysis in the FIRST BREATH Trial, NRP training in the DRC had no demonstrable effect on neonatal or perinatal mortality. The lack of benefit of NRP training is most likely attributable to the fact that the strategies for resuscitation recommended in the ENC program reach the limits imposed by the environment of most deliveries in rural DRC. Although the NRP includes a more regimented approach to resuscitation, benefit beyond that achieved by ENC training may depend upon the availability of more extensive resources at the site of delivery.

The effect of ENC training has been evaluated in other studies in low resource countries. Two studies evaluated the benefits of incorporation of ENC training of professional birth attendants in Sri Lanka[12,13]. Using an active baseline study design similar to ours, ENC training resulted in an increased utilization of ENC practices, but no change in mortality. These studies apparently did not include a strategy for re-enforcement of training, which may account for the lack of impact on mortality. By contrast, among midwives practicing in urban health clinics in Zambia, ENC training improved cognitive knowledge and practical skills[14], and these changes translated into reduced early neonatal and perinatal mortality[15]. In this environment, ENC training appeared to provide less benefit among mothers with primary education only compared to mothers with more education. This finding implies that the impact of training of birth attendants is not dependent solely upon improved skills of birth attendants. ENC training may also improve care provided by mothers which impacts neonatal mortality. Of importance in interpreting the results of our study is the fact that few women in the study communities in the DRC had more than primary education. It is possible that greater benefit might have been achieved in urban areas where the general education of the public is higher.

Our study has a number of strengths. It was population based, and used pregnancy and birth registries to capture all births during the study period, although we cannot be certain all births were included because the registries may have been of variable quality. Birth attendants received intense instruction in the differentiation between stillbirths and live born infants. Therefore, we believe that we have accurately assessed the contribution of stillbirth to perinatal mortality. Follow up rates to seven days approached 100%. To our knowledge, this is the largest population-base study conducted in rural areas of the DRC. Because the demography and medical resources in the area in which the study was conducted are similar to other rural areas of the DRC, we assume that the results are generalizable to those areas, and perhaps other low resource countries with similar environments.

An important limitation in this study was the use of pre-post data as a reference for determining the effect of the ENC program. To minimize the impact of this aspect of study design, all training, except ENC and NRP training, was conducted before the initiation of any data collection. This active baseline study design ensured, to the extent possible, that observed changes in mortality resulted from changes in practice rather than changes in the quality of data[16]. In addition, our most informative analysis, examination of the effect of time after ENC training on mortality, was not entirely dependent upon data collected during the pre-ENC training period as the rate during this period represented a single time point only in the analysis. A second limitation was the late entry of two communities into the study. The inclusion of data from these communities may have reduced the apparent beneficial effects of ENC training because of the truncated time between training and the end of the study period in these communities. Another limitation was that data collection was performed by the birth attendants who implemented the intervention. Reliable data collection was ensured through close supervision of the birth attendants by the CCs.

## Conclusions

We conclude that training birth attendants using the ENC program may reduce perinatal mortality by reducing neonatal deaths during the first seven days after birth. However, this benefit may not be achieved immediately after training. A period of utilization and re-enforcement of training may be necessary before a decline in mortality occurs. Reduced mortality following training can be expected among births attended by both professionals and non-professionals, including TBAs. As in the DRC, ENC training has the potential to be a low cost, high impact intervention in many other low resource countries, where deliveries are commonly attended by TBAs. A train-the-trainer model appears to be effective; the use of this strategy may minimize the impact of the isolation of many rural communities and their distance from sites of traditional medical education. Our findings suggest that implementation should include a strategy for re-enforcement following the initial training.

## List of abbreviations

CC: Community Coordinator; CI: confidence interval; DRC: Democratic Republic of Congo; ENC: Essential Newborn Care program; GEE: generalized estimating equation; NRP: Neonatal Resuscitation Program; RR: risk ratio; TBA: traditional birth attendant; WHO: World Health Organization.

## Competing interests

The authors declare that they have no competing interests.

## Authors' contributions

RM, the principal and corresponding author, drafted the paper, and provided technical assistance (training, supervision and project monitoring) and management of the field staff in charge of the study data collection. CE provided expert technical assistance to the country study team and revised the manuscript. JD collaborated closely with RM on technical and managerial aspects of the study; he revised the manuscript. JG managed the data collection on a daily basis, providing direct oversight to birth attendants in the field. He also provided input on data analysis. AT, the Senior Foreign Investigator of the First Breath Study in the DRC, revised the manuscript. RK, worked in-country at the outset of the study, and helped develope the infrastructure of study team. She also assisted with training and study oversight. EM helped with data organization and analysis, and revised the manuscript. JM performed data analysis and revised the manuscript. DW developed the time-dependent statistical models and assisted with other aspects of data analysis. WC, Principal Investigator of the international First Breath Trial, revised the manuscript. LW provided technical and managerial support on behalf of the NIH and the Global Network for Women's and Children's Health Research. She also revised the manuscript. CB, Principal Investigator of the First Breath Study for the DRC, supervised the conduct of the study and revised the manuscript. All authors read and approved the final manuscript.

## Pre-publication history

The pre-publication history for this paper can be accessed here:

http://www.biomedcentral.com/1741-7015/9/93/prepub
